# Dose-Effect Determination of a Neuroprotector Fraction Standardized in Coumarins of *Tagetes lucida* and Bioavailability

**DOI:** 10.3390/pharmaceutics15030967

**Published:** 2023-03-17

**Authors:** Anislada Santibáñez, Maribel Herrera-Ruiz, Manasés González-Cortazar, Pilar Nicasio-Torres, Ashutosh Sharma, Enrique Jiménez-Ferrer

**Affiliations:** 1 Centro de Investigación Biomédica del Sur, Instituto Mexicano del Seguro Social, Argentina No. 1 Col Centro, Xochitepec 62790, Morelos, Mexico; 2 School of Engineering and Sciences, Tecnologico de Monterrey, Plant Innovation Lab, Av. Epigmenio González No. 500, San Pablo 76130, Queretaro, Mexico

**Keywords:** pharmacokinetics, pharmacodynamics, 7-*O*-prenylscopoletin (PE), scoparone (SC), dimethylfraxetin (DF), herniarin (HR), 7-*O*-prenylumbelliferone (PU), neuroprotector

## Abstract

Neurodegeneration has been associated with chronic inflammation states in the brain. For this reason, attention has been directed to drugs indicated as anti-inflammatory as possible therapies for the treatment of said conditions. *Tagetes lucida* has been widely used as a folk remedy in illnesses associated with the central nervous system and inflammatory ailments. Among the compounds that stand out in the plant against these conditions are coumarins, such as 7-*O*-prenyl scopoletin, scoparone, dimethylfraxetin, herniarin, and 7-*O*-prenylumbelliferone. Therefore, the relationship between the therapeutic effect and the concentration was evaluated through pharmacokinetic and pharmacodynamic studies, including vascular permeability evaluation by blue Evans and pro- and anti-inflammatory cytokines quantification, under a neuroinflammation model induced by lipopolysaccharide by the oral administration of three different doses (5, 10, and 20 mg/kg) of a bioactive fraction of *T. lucida*. In the present study, it was found that all doses showed a neuroprotective and immunomodulatory effect, although the doses of 10 and 20 mg/kg were able to exert their effect for a longer time and to a greater extent. The protective effects of the fraction may be mainly associated with the DR, HR, and SC coumarins due to their structural profile and plasmatic and brain tissue bioavailability.

## 1. Introduction

Neurodegenerative diseases (ND) are characterized by progressive dysfunction, as well as neuronal and functional loss of the Central Nervous System (CNS) [1]. Neurodegenerative diseases currently affect more than 50 million people worldwide and represent the leading causes of disability and death among the elderly population [2]. Nowadays, an optimal treatment has not been developed to delay, reverse, or even avoid the neurodegeneration process [3]. Largely because the symptoms and pathological processes are varied in each disease.

However, among the developments of pharmacological therapies, it has been determined that neuroinflammation is a common factor in the establishment and progression of neurodegeneration due to defects caused in the regulatory pathways of the CNS [4,5]. As in any inflammatory process, the response generated by specific CNS cells, such as microglia, has a protective effect by maintaining optimal regulation within the brain [6]. However, under certain pathological conditions or harmful stimuli against the CNS, these cells are activated, giving rise to the presence of two phenotypes commonly characterized by its neurotoxic (M1) or neuroprotective (M2) effects [7,8].

The M1 phenotype is related to the production of harmful mediators as proinflammatory cytokines, such as tumor necrosis factor-alpha (TNF-α), a highly inflammatory cytokine that plays an essential role in mediation of the transcription of anti-apoptotic and anti-inflammatory genes that regulate CNS activities [9,10,11]. While its M2 counterpart exerts an anti-inflammatory effect by releasing cytokines such as IL-10, M1 plays a key role in the resolution of inflammation on the CNS because the receptor for this cytokine is expressed in microglia to produce its activity locally [6,7,12]. The expression of these pro- and anti-inflammatory mediators released in response to microglia activation are used as markers of progression of neuroinflammation in neurodegenerative diseases [7,13].

On the other hand, the blood–brain barrier (BBB) allows the regulation of the entry of molecules from the bloodstream into the brain tissue through selective transporters. Some cell signaling proteins such as cytokines can cross the BBB from the periphery to reach the brain. Entry of TNF-α into the BBB damages the tight integrity of the barrier, leading to exacerbated levels of the cytokine that trigger cyclical processes of inflammation and apoptosis that ultimately lead to CNS disease states [10,11,14].

Local or peripheral administration of LPS, an endotoxin purified from the cell wall of Gram-negative bacteria, activates immune cells that trigger a systemic and peripheral inflammatory response through different mechanisms of action [15]. First, it has its downstream effect by binding to Toll-like receptor 4 (TLR4); these receptors are abundantly expressed in microglia, leading to activation of nuclear factor-κB (NF-κB), inciting the synthesis and release of proinflammatory cytokines mediated by myeloid differentiation adapter 88 (MyD88). LPS also induces amyloidogenesis by activation of β-secretase (BACE1) and γ-secretase, which consequently increases the levels of β-amyloid peptide (Aβ) after hydrolysis of the amyloid precursor protein (APP). In addition, LPS generates increased expression of microRNA-155 (miRNA-155), which downregulates the suppressor of cytokine signaling (SOCS1), a negative regulator of cytokine signaling, stimulating the inflammatory process [16]. It has been generally established that the peripheral administration of LPS in adult and old mice at high doses produces an exacerbated neuroinflammatory response, therefore offering an ideal model for the evaluation of new anti-inflammatory drugs for CNS that may exert neuroprotective effects against neuroinflammation and cognitive decline in mice through possible modulation of TLR4/MYD88/NF-κB, miRNA-155/SOCS-1, and cAMP/phosphorylated CREB (pCREB) signaling pathways [16,17,18].

Among the main sources of new anti-inflammatory therapeutic agents are natural products derived from plants. *Tagetes lucida*, a plant of the Asteraceae family, has well-documented ethnopharmacological reports that test its activity in biological models as inflammatory inhibitors [19,20]. At the same time, the chemical components associated with the effect have been determined where coumarins stand out. In the *T. lucida* hexanic extract coumarins 7-O-prenylscopoletin (PE), scoparone (SC), dimethylfraxetin (DF), herniarin (HR), and 7-O-prenylumbelliferone (PU) had shown a potential anti-inflammatory activity [19]. Whereby, this type of compounds represents potential therapeutic agents in the treatment of neuroinflammation.

During the new herbal drug development, pharmacokinetic (PK) studies become essential to providing information on the prediction of the distribution, metabolism, and excretion of the chemical participants. As has been tested with the bioactive fraction obtained from the *T. lucida* hexanic extract, administered at a dose of 10 mg/kg, carried out in our previous study [21]. Nonetheless, it is important to establish the relationship between different doses and the exposure of the molecules at the site of action to evaluate the properties and therapeutic efficacy, through biological tests, that the herbal drug has at different concentrations to the binding target [22,23,24].

It should be noted that these studies are particularly complex in tissues related to neurodegenerative diseases, essentially due to the presence of the BBB, one of the main limitations in getting bioactive compounds to reach the site of action [22]. For this, the evaluation of vascular permeability with Evans blue dye along with cytokines quantification will determine the integrity of the BBB by inhibiting the damage caused by LPS associated with three different regimen doses concentrations and the PK behavior of bioactive compounds to determine the course temporal dose effect of the bioactive fraction derived from hexanic extract of *T. lucida*.

## 2. Materials and Methods

### 2.1. Chemicals

Rutin (internal standard, IS), trifluoroacetic acid (TFA), lipopolysaccharide (LPS), blue Evans dye (BE), sodium chloride (NaCl), sodium dihydrogen phosphate (Na_2_HPO_4_), were purchased from Sigma-Aldrich (St. Louis, MO, USA). HPLC-grade solvents: acetonitrile, methanol, and water, were acquired from Tecsiquim (Mexico, Mexico), Tween 20, potassium chloride (KCl), potassium dihydrogen phosphate (KH_2_PO_4_), and reagent-grade hexane and ethyl acetate were purchased from Merck (Darmstadt, Germany).

### 2.2. Standardization of the Bioactive Fraction of Tagetes lucida

A hexanic extract from *Tagetes lucida* was obtained as described in our previous work [21]. Briefly, dry milled plant material collected in Xochitepec, Morelos, Mexico identified with the voucher No. 2081 [25], was macerated for 24 h with hexane in a 1:3 (*w*/*v*) proportion by three times. Then, the extract was subjected to open column chromatography with five elution systems (hexane:ethyl acetate) increasing the polarity by 5% for each system (250 mL), starting in 100% hexane.

Finally, it was established that the standardized coumarin content in mg/g of extract was equal to previously reported, using the same HPLC parameters that were described in the mentioned work [21]. Briefly, a Waters 2695 separation equipment coupled to a UV-VIS detector (Waters 2696) was used to carry out a 30 min run with a gradient elution composed of an aqueous solution of trifluoroacetic acid at 0.5% and acetonitrile (Table S1). The injection volume is 10 µL per sample at a flow of 0.9 mL/min of the mobile phase. The data were recorded at a wavelength (λ) of 330 nm and were processed with Empower Pro 3.0 software (Waters, MA, USA).

### 2.3. Preparation of Administration Drugs and Solutions

Three aqueous solutions from active fraction from *T. lucida* with 1% Tween-20 were prepared at concentrations of 0.5, 1.0, and 2.0 mg/mL to be used as treatments.

Likewise, a 200 µg/mL solution of lipopolysaccharide (LPS) and another of 0.5% Evans blue dye were prepared independently, contained in sterile phosphate-buffered saline (PBS) with 1% Tween-20. PBS contained NaCl (8.06 g/L), KCl (0.22 g/L), Na_2_HPO_4_ (1.15 g/L), and KH_2_PO_4_ (0.20 g/L), adjusted to pH 7.4.

### 2.4. Animals

Male ICR strain mice between 8 to 10 weeks of age, provided by the Century XXI Medical Center animal facility, with a weight range of 35 ± 5 g were used throughout the experiments. Six mice per cage were placed and kept under the conditions of the vivarium with cycles of 12 h of light/darkness (07:00 to 19:00 h) at 25 °C. Access to food (Rodent Laboratory Diet pellets, Harlan) and water was allowed ad libitum up to 12 h before the start of the evaluations. The mice were adapted to the laboratory environment three weeks prior to their use in the experiments.

The studies were carried out in accordance with the Official Mexican Standard NOM-062-ZOO-1999: Technical specifications for the production, care, and use of laboratory animals [26]. The Local Health and Ethics Research Committee of the Instituto Mexicano del Seguro Social (IMSS) approved the present project on 16 August 2021 (Registration number: R 2021-1702-009).

### 2.5. Pharmacokinetic and Brain Distribution Study

**Figure 1:**
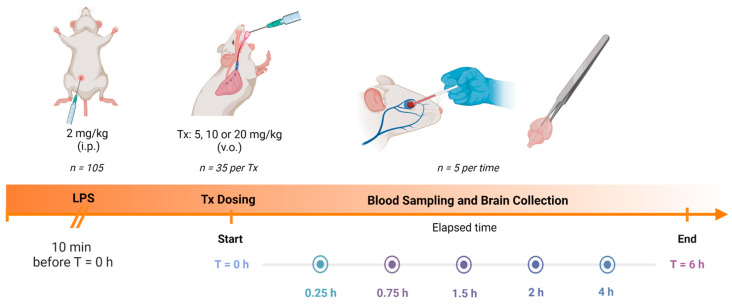
Overview of the pharmacokinetic and brain distribution study.

In Figure 1 is summarized the pharmacokinetic and tissue distribution study of the coumarins PE, SC, DF, HR, and PU contained in the bioactive fraction of *T. lucida,* where a total of 105 mice were used.

Ten minutes before starting the study, all mice underwent an acute neuroinflammation process caused by the intraperitoneal (i.p.) administration of the LPS solution at 2 mg/kg. Once the time had elapsed, the mice were divided in three groups according to the different administered doses: 5, 10, or 20 mg/kg of the fraction previously prepared, based on the effect offered by these coumarins in CNS [19,25].

Finally, blood and tissue samples from mice (*n =* 5 per time), were obtained and processed at 0, 0.25, 0.75, 1.5, 2, 4, and 6 h post-dosing, as indicated in the following section for their further analysis.

#### 2.5.1. Samples Collection and Processing

First, 500 µL of blood samples were collected at the established times from the retroorbital sinus of the mice in heparinized Eppendorf tubes. The plasma was separated, as soon as possible, by centrifugation at 3500 rpm for 5 min and the plasma was separated into new tubes stored at −70 °C until use.

Immediately after obtaining the blood samples, the mice were euthanized in a euthanasia chamber (10 cm × 20 cm × 5 cm) containing cotton pads and gauze soaked with diethyl ether (10 mL), as well as subsequent cervical dislocation [26]. Once the death of the animal was verified, the brain was dissected. The organs were rinsed with saline solution, to avoid exogenous contaminants or interfering in the quantification, and immediately placed on ice. The fresh weight of the brains was recorded, later subjected to a freeze-drying process (LABCONCO ^®^, Kansas City, MO, USA), grounded and finally dry weighed.

#### 2.5.2. Samples Coumarins Extraction and Analysis

Each grounded organs were suspended in methanol in 1:1 dry weight:volume ratios for 24 h and centrifugated at 14,000 rpm for 7 min. Finally, the supernatants were collected in clean tubes and stored at −70 °C until use.

To 100 µL of the plasma or tissue homogenate samples previously obtained, 300 µL of acetonitrile with IS (10 µg/mL) were added and placed in a vortex for 3 min. Subsequently, 200 µL of dichloromethane were added and mixed for 5 min. The aggregate was centrifuged at 14,000 rpm for 10 min and the organic layer was separated and filtered into new tubes to be brought to dryness. For HPLC-UV analysis, samples were resuspended in methanol and 10 µL of sample was injected into the system, as indicated in HPLC-DAD-UV handling conditions section by Santibáñez et al. [21].

#### 2.5.3. Pharmacokinetic and Brain Distribution Analysis

Following the methodology indicated for PK analysis in our study previously mentioned, the parameters maximum plasma concentration (C_max_), time to reach the maximal concentration (Tmax), half-life time (t_1/2_), area under the concentration–time curve to 6 h (AUC_0–6_) and to infinity (AUC_0–∞_), mean residence time (MRT), and the observed oral clearance (CL/F) of PE, SC, DF, HR, and PU in plasma were calculated by PKSolver software [27] using a non-compartmental model for each of the three doses, as well as the quantification of the coumarins content in liophilized brains by HPLC system.

### 2.6. Vascular Permeability Evaluation

**Figure 2:**
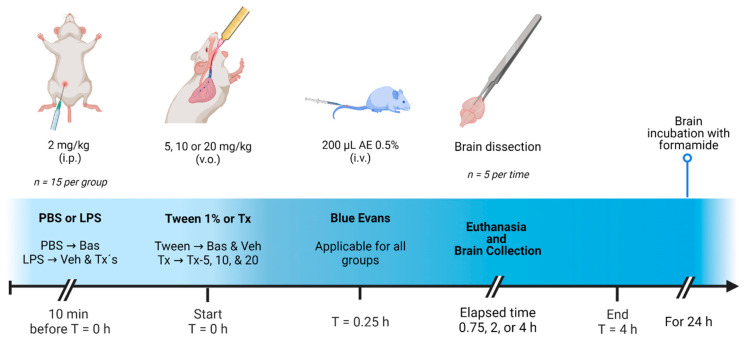
General scheme of vascular permeability evaluation.

The integrity of BBB after an acute inflammation damage was evaluated in 75 mice equally divided in the groups: Basal (Bas), vehicle (Veh), and three treatment groups (Tx) for each dose of 5 mg/kg (Tx-5), 10 mg/kg (Tx-10), and 20 mg/kg (Tx-20), as shown in Figure 2.

To begin the evaluation, mice, except for those in the Bas group, get an i.p. administration of LPS at 2 mg/kg. After 10 min of damage induction, the Bas and Veh groups were administered orally with a Tween 1% aqueous solution and the remaining mice were administered with the corresponding dose of coumarin treatment. Fifteen minutes later, 200 µL of a PBS solution was added with 0.5% Evans blue were perfused through the lateral tail vein. Finally, the mice were euthanized, as described before, at 0.75, 2 and 4 h post- administration and the brains of mice were immediately dissected, rinsed with saline solution, weighted, and incubated in a 55 °C water bath in Eppendorf tubes containing 500 µL of formamide for 24 h.

Once the time had elapsed, the brains were centrifuged at 14,000 rpm for 7 min and 300 µL of the supernatant were recovered in 96-well microplates for analysis in the spectrophotometer at λ = 620 nm, using formamide as blank. A quantitative analysis of the content of BE in the tissues was carried out with a calibration curve based on external standard method in formamide.

### 2.7. Cytokine Quantification

**Figure 3:**
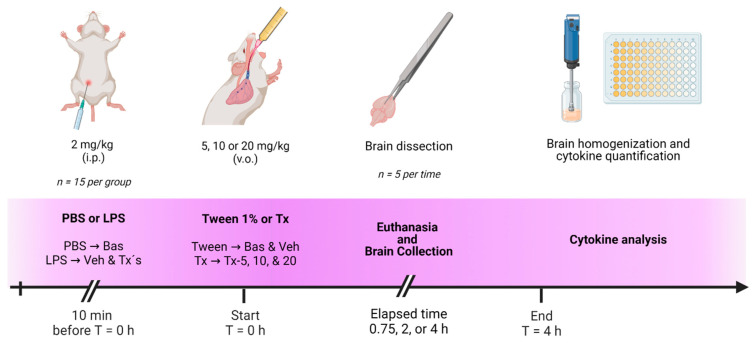
Overview for cytokine analysis.

For cytokine analysis, sketched in Figure 3, the same experimental design as in vascular permeability assay was used. After dosing profiles and evaluation times were completed, the brains were dissected and individually homogenized at a 5:1 weight:volume ratio with a PBS solution at pH 7.4 with 1% phenyl methyl sulfonyl fluoride (PMSF), using an ULTRA-TURRAX T25 basic polytron at 6500 rpm. After homogenization, the samples were centrifuged at 14,000 rpm for 7 min, the supernatant was recovered for cytokine quantification by the ELISA method using cytokine kits (BD Biosciences, Franklin Lakes, NJ, USA), according to the manufacturer’s instructions for IL-10 and TNF-α: Mouse IL-10 ELISA Set (Cat. No. 555252) and Mouse TNF Mono/Mono ELISA Set (Cat. No. 555268), respectively.

### 2.8. Statistical Analysis

Each experiment was conducted using five individuals per group. The resulting data were expressed as mean ± standard error (SEM). Afterwards, they were analyzed by using an Analysis of Variance (ANOVA) followed by the post hoc Dunnet test (* *p* < 0.05 and ** *p* < 0.01). Statistical analysis was performed with IBM SPSS Statistics ver. 25.0 software program (SPSS Inc., Chicago, IL, USA).

## 3. Results and Discussion

### 3.1. Chemical Composition of the Bioactive Fraction of Tagetes lucida

The species of *Tagetes lucida* is rich in content of terpenes, phenolic acids, flavonoids and coumarins, mainly [19,20,28,29,30,31]. These constituents offer a wide range of biological activities due to their antidepressant, antibacterial, antinociceptive, antispasmodic, antihypertensive, and specially antioxidant and anti-inflammatory properties [19,20,28,32,33,34,35,36]. Due to its valuable pharmacological properties, this plant can be used as a potential source of novel herbal medicines to treat conditions related to CNS diseases, such as neurodegenerative diseases, which have related their etiology to neuroinflammation [37,38].

**Figure 4:**
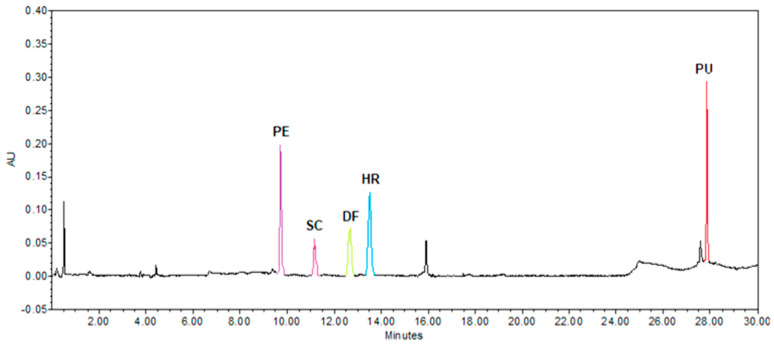
Chromatogram of the bioactive fraction of *T. lucida* at λ = 330 nm. PE = 7-*O*-prenylscopoletin, SC = scoparone, DF = dimethylfraxetin, HR = herniarin, and PU = 7-*O*-prenylumbelliferone.

The bioactive fraction for oral administration, with a high content of anti-inflammatory coumarins, obtained from the hexane extract of *T. lucida* was analyzed at λ = 330 nm by HPLC-UV (Figure 4).

In the present work, a fraction rich in coumarins was used: PE, SC, DF, HR, and PU, with previously proven anti-inflammatory activity for each of these components [19]. However, one of the main limitations that arise during the development of medicines obtained from natural sources is the poor standardization of bioactive components [23]. The RT and UV spectra were compared with the PE, SC, DF, HR, and PU standards obtained by prior purification in the laboratory to determine which corresponded to the coumarins of interest [21]. The RT values, maximum absorbance (λ_max_), and the concentrations of each coumarin quantified in the fraction are shown in Table 1.

### 3.2. PK of Different Doses of Active Fraction of T. lucida

**Figure 5:**
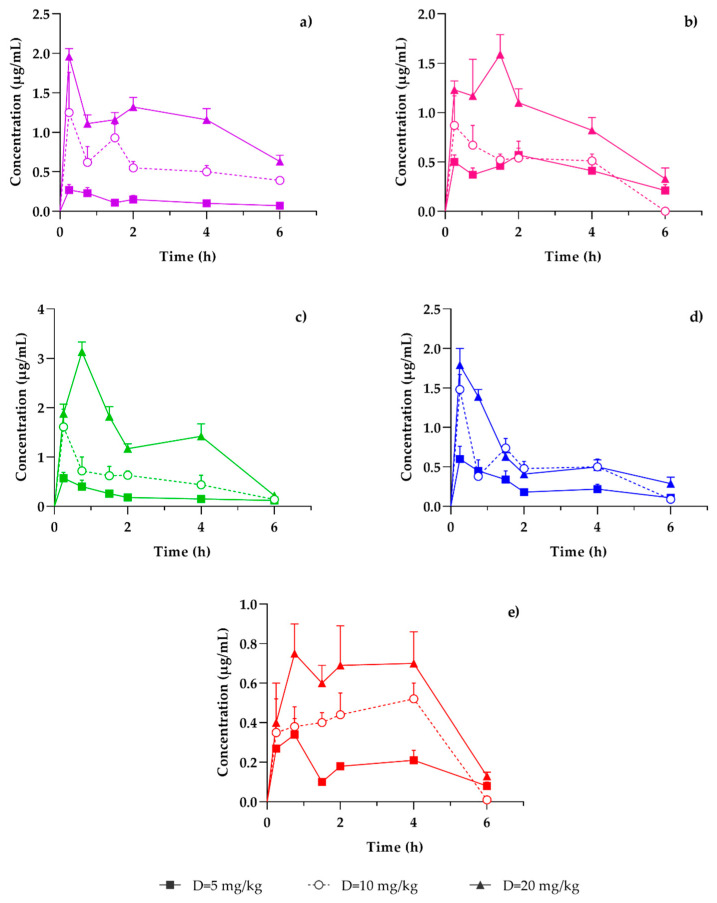
Concentration-time profile of coumarins in plasma samples: (**a**) PE, (**b**) SC, (**c**) DF, (**d**) HR, and (**e**) PU, after a dose administration of 5 (-■-), 10 (-○-) [21], or 20 mg/kg (-▲-) of the hexanic extract of *Tagetes lucida*, in ICR mice LPS-damaged. Results are presented as mean ± SEM (*n* = 5). As observed in Figure 4, a dose-dependent concentration behavior was presented for the five coumarins. However, the concentration-time profile presented differences in the maximum concentration values; PE and HR showed their maximum concentration at 30 min. DF and PU presented their maximum concentration at min 60, while SC presented it at 120 min.

**Figure 6:**
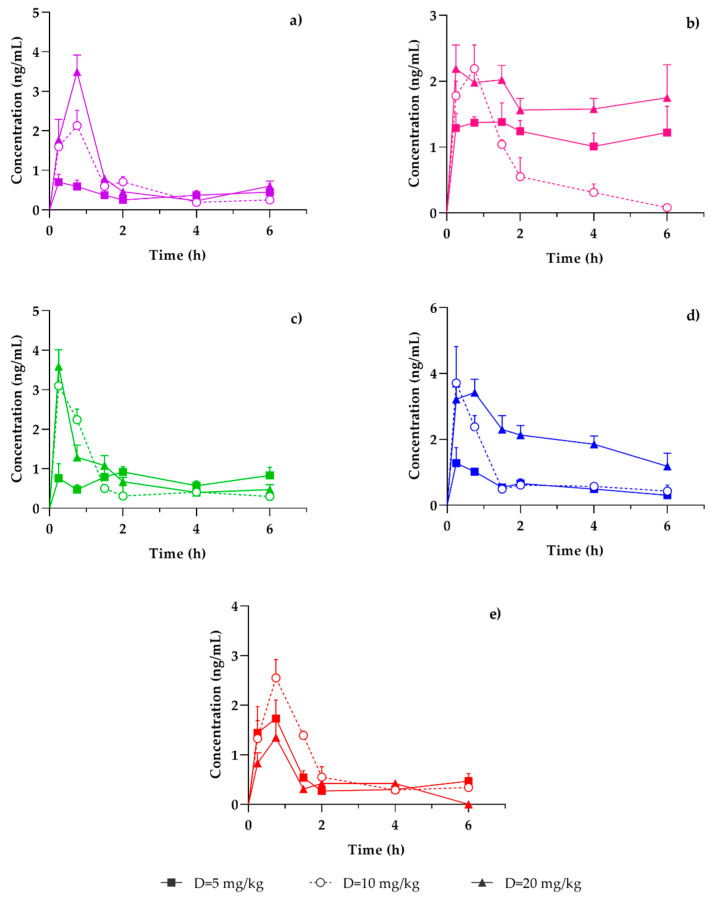
Concentration-time profile of quantified coumarins in the brain: (**a**) PE, (**b**) SC, (**c**) DF, (**d**) HR and (**e**) PU. After administration of three doses of the hexanic extract of *Tagetes lucida* 5 (-■-), 10 (-○-) [21], or 20 mg/kg (-▲-), in ICR mice exposed to damage by the administration of LPS. Results are presented as mean ± SEM (*n* = 5).

Applying a validated method for the quantification of coumarins by HPLC, which has previously been reported [21], samples from mice that were administered three different doses, 5, 10 [21], and 20 mg/kg, of *T. lucida* bioactive fraction, were analyzed to determine the temporal course of the variation in the concentration of PE, SC, DF, HR and PU was evaluated, both in plasma (Figure 5) and in the brain (Figure 6).

Consistent with the neuroprotective effect of *T. lucida*, the study of variation in the plasmatic concentration of active coumarins was carried out in mice previously exposed to neuro-inflammation, secondary to the administration of LPS, where the PK parameters were obtained (Table 2).

Comparing the maximum concentration values of the coumarins, presented in Table 2, with the administered dose (C_max_/dose), we obtain the following sequence of ratios: DF >> SC > PE ≈ HR > PU, (DF = 11.87, SC = 6.89, PE = 4.70, HR = 4.54, PU = 2.16). What differs from the administered dose PE ≈ PU > HR > SC ≈ DF.

Regarding the above, the importance of chemical groups that substitute in the aromatic ring of coumarins is notable. DF is the one that was administered in a lower dose according to the standardization of the active fraction (13.18 mg/g) and DF is the one that reached the highest index of Cmax/dose; structurally what distinguishes DF is the presence of 3 methoxyls at C-6, C-7, and C-8. Secondly, in the sequence, it is SC, and this coumarin only has 2 methoxyls at C-6 and C-7. HR has a lower value of the aforementioned index and only presents a methoxyl at C-7, while PE has a similar value of the index with an isoprenoxyl group at C-7 and methoxyl at C-6. Considering the PU index, which only has isoprenoxyl at C-7, it indicates the importance of the substituent size and position.

### 3.3. Brain Distribution of Coumarins in T. lucida

Figure 6 shows the variation tissue concentration profile of the coumarins PE, SC, DF, HR, and PU, of the same mice in which the plasmatic bioavailability of the coumarins present in the fraction of *T. lucida* was analyzed. Unlike the dose-dependent behavior of C_max_, obtained in the determinations of the plasmatic concentration of said coumarins, only PE clearly showed this behavior (Figure 6a). The most evident contrast was PU, where the C_max_ of a lower dose (10 mg/kg) of hexane extract showed a higher C_max_ than was observed with the 20 mg/kg dose (Figure 6e). With SC, DF, and HR, very similar C_max_ values were reached, with the 10 and 20 mg/kg doses (Figure 6b–d).

When the ratio Cmax/dose was calculated (DF = 14.03, HR = 9.34, PE = 8.88, SC = 7.51, PU = 6.63), the following comparative sequence of values was observed: DF >>HR ≈ PE > SC > PU. Once again, the categorical presence of the three methoxyl substituents present in the molecule was corroborated at C-6, C-7, and C-8 to define the greater bioavailability of in brain tissue, noting that in this case the blood–brain barrier (BBB) is another frontier that must be crossed to reach the brain. However, HR (C-6) resulted in a higher bioavailability than SC (C-6 and C-7), where the bioavailability of coumarins gradually decreased as the number and position of methoxyl substituents were eliminated. However, we can consider that this trend is maintained, to the extent that the bioavailability values of HR and SC are very close.

### 3.4. Vascular Permeability Evaluation by Blue Evans Dye in Brain

**Figure 7:**
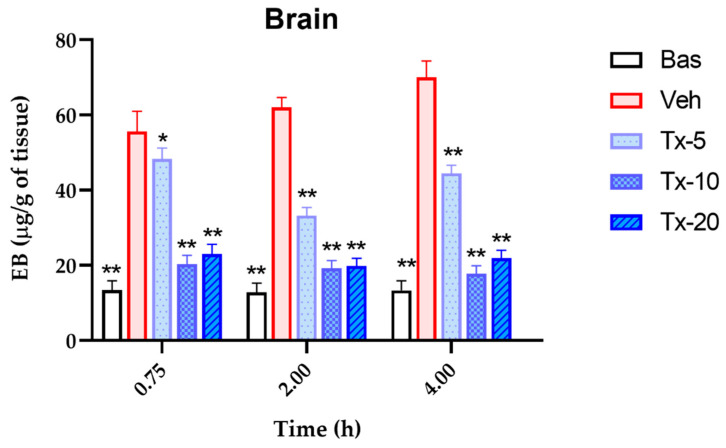
Evans blue concentration-time profile quantified in the brain to measure extravasation of plasma fluid in brain tissue. The brain of mice exposed to neuroinflammation by administration of LPS and treated with different doses of *T. lucida* bioactive fraction: 5, 10, and 20 mg/kg. Results are presented as mean ± SEM, ANOVA post hoc Tukey comparing against damage group (Veh). (* *p* < 0.05, ** *p* < 0.01); (*n* = 5).

Figure 7 shows the kinetics of extravasation of plasma fluid into brain tissue, measured by the release into the extravascular space of Evans Blue. Neuronal damage from the exposure of the mice to LPS by systemic administration is evident, as shown by the size of the vehicle (Veh) bar with respect to that observed in the basal (Bas) condition. In the same way, the increase in extravasation is dependent on the exposure time, as demonstrated by the increasing trend of the vehicle bars, as the exposure time progresses. Additionally, treatments (Tx) with the bioactive fraction standardized in coumarins counteract the extravasation of plasmatic fluid secondary to systemic inflammation. This decrease is dose-dependent, which allowed us to calculate the associated pharmacological constants. At 0.75 h, E_max_ = 92.60% and DE_50_ = 10.32 mg/kg; at 2:00 h, E_max_ = 86.21% and ED_50_ = 4.39 mg/kg; and 4:00 h, E_max_ = 100 % and ED_50_ = 8.79 mg/kg.

It is noteworthy that the effect of LPS on the increase in extravasation of plasmatic fluid depends on the exposure time. This is due to two considerations, firstly to paracellular mechanisms, and secondly to transcellular processes; the first of these result from the structural and functional alterations that LPS causes on endothelial cells (EC) that participate in the BBB [39]. Under homeostatic conditions, the BBB maintains conditions of isolation from potential aggressive stimuli from the environment [40].

The low permeability of the BBB, dependent on paracellular mechanisms, is exerted in principle by tight junctions (TJs). These consist primarily of the densely distributed transmembrane protein claudin (especially claudin-5), occluding, tricellulins, junctional adhesion molecules, and intracellular support proteins such as zonula occludens (ZO). TJs seal the paracellular pathway, significantly reducing the penetration of polar solutes from the plasma into the interstices of the brain and are responsible for the integrity of the BBB [41,42].

On the other hand, there are also adherent junctions (AJ) formed by intercellular cadherin and intracellular catenin and are also responsible for sustaining BBB function. It has been reported that LPS impairs the BBB by significantly increasing the permeability of solutes from the peripheral circulation to the cerebral space by eliminating TJ and AJ by decreasing their gene expression and altering distribution [43]. This paves the way for harmful chemicals that bypass the BBB and alter the functions of the CNS. It has also been described how LPS causes a disruption of the BBB by increasing the expression of C-X-C Motif Chemokine Receptor 2 (CXCR2) in brain EC in a time-dependent manner, where endothelial actin polymerization is involved [39].

Another protein involved in LPS-mediated damage to TJs is matrix metalloproteinase (MMP), especially MMP-9, which participates in TJ degradation and therefore increases BBB permeability [44]. On the other hand, LPS can stimulate the expression of NF-κB, which is a key regulator of gene expression of proteins related to the inflammatory process; this results from the binding of LPS by binding to the Toll-like receptor 4 (TLR4) [45,46,47]. Thus, LPS can destroy BBB by reducing the level of occludin through the TLR4/NF-kB pathway [45]. In addition, it has been described as LPS can activate RhoA as well as downstream NF-κB, and lead to increased phosphorylation of myosin light chain (MLC), which ultimately causes a decrease in the expression of TJs proteins, claudin-5 and ZO-1. Therefore, NF-κB play a determining role in TJ damage in LPS-associated inflammatory responses, which ultimately impairs BBB function [48,49].

In the literature, it is mentioned that biological interactions, including those between drugs and their receptors, establish a rapid equilibrium, such that changes in the concentration of the free ligand (drug) are immediately reflected in an alteration in the amount of bound ligand (drug-receptor complex). Although, there is a history that almost 80% of the FDA-approved drugs operate through a non-equilibrium mode of action and therefore the activity of the drug in vivo is controlled by both the thermodynamics and the kinetics of drug-receptor interactions. This is supported by the fact that many drugs dissociate slowly from their drug targets, which supports that the kinetics of drug-receptor interactions provides a more realistic view of the drug’s mode of action [50].

### 3.5. TNF-α and IL-10 Cytokine Quantification

**Figure 8:**
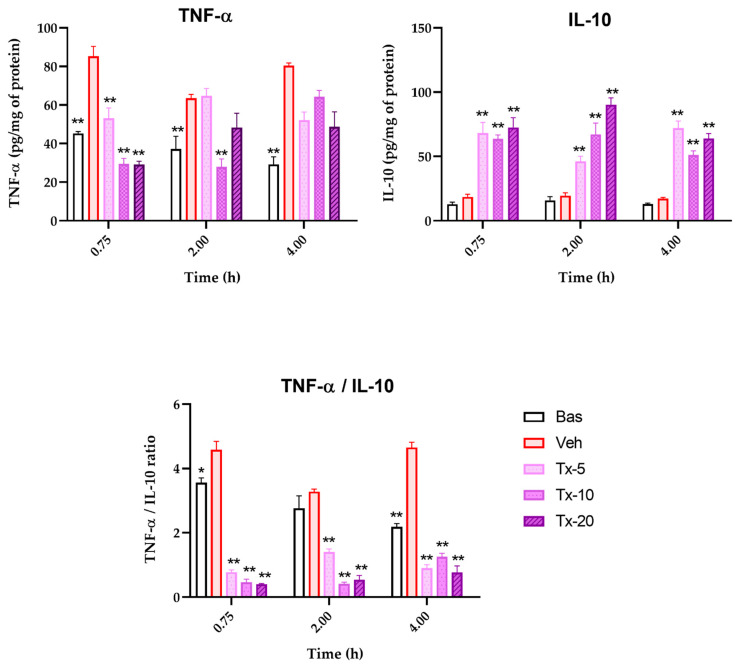
Concentration-time profile of TNF-α and IL-10 quantified in brain tissue to measure the evolution of the inflammatory process. The brain of mice was exposed to neuroinflammation by administration of LPS (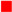) and treated with different doses of *T. lucida* fraction: 5, 10, and 20 mg/kg. Results are presented as mean ± SEM, ANOVA post hoc Tukey comparing against damage group (Veh); (* *p* < 0.05, ** *p* < 0.01); (*n* = 5).

In Figure 8, the kinetics of TNF-α and IL-10 production and release are shown. In the first panel (TNF-a), it is observed that the differential between the TNF- α released by the Veh group and the Bas group, present a tendency to increase the value of the differential. With the different doses of the bioactive fraction treatments, a tendency to decrease the concentration of this proinflammatory cytokine is observed.

The following panel presents the behavior of IL-10. It is notable that Veh and Bas groups show similar levels so that the neuroinflammation process results from a basic response of the production and release of IL-10. In contrast, the treatments stimulate IL-10 release several times, indicating that the control of neuroinflammation is probably secondary to IL-10-driven modulation. In order to estimate the degree of immunomodulation, the TNF-a/IL-10 index is calculated, thereby generating the graph of the third panel. It was observed that the value of the index shows a remarkable dose-dependent behavior. This allowed us to calculate the associated pharmacological constants. The Emax indexes in each of the chosen times, 0.75, 2.00, and 4.00 h, were 4.44, 3.36, 4.24, respectively. While the EC50 values were 2.04, 4.33, and 0.2 mg/kg in the same order.

The inflammatory stimulus of LPS in the CNS results from binding to Toll-like receptor 4 (TLR4), present in large quantities in microglia, which causes phosphorylation of nuclear factor-κB (NF-κB), which leads to the production of proinflammatory cytokines via the primary myeloid differentiation adapter 88 (MyD88) response [16]. The neurotropic activity of scoparone has recently been described in models of neurodegeneration, by administration of LPS. However, SC did not reverse LPS-mediated cognitive impairment, suggesting no central anti-inflammatory effects. On the contrary, the acute administration of scoparone led to a very strong and unexpected increase in arachidonic acid and prostaglandins, but also N-acylethanolamines and a concomitant decrease in 2-arachidonoyl glycerol (2-AG). The latter has been implicated in anxiety and is currently being studied in the context of stress-related conditions [51].

Furthermore, studies have shown increased expression of microRNA-155 (miRNA-155) after LPS administration. MiRNA-155 downregulates the suppressor of cytokine signaling (SOCS1) in such a way that LPS by this mechanism stimulates the inflammatory process [16]. Therefore, the neuroprotective effect of coumarins present in the active fraction of *T. lucida* is established by the possible modulation of TLR4/MYD88/NF-κB, miRNA-155/SOCS-1.

## 4. Conclusions

The chemical characterization of the active fraction was achieved, since the concentration of five coumarins (PE, SC, DF, HR, and PU) was determined and that when they were administered orally in increasing doses, the sequence of bioavailability values was established, where DF, the highest value, and PU, the lowest value, are both in plasma and in the brain. Regarding the pharmacological effect, the neuroprotection against the damage caused by LPS of the fraction standardized in coumarins was determined, by reducing vascular extravasation and generating an environment of immunomodulation, associated with the decrease in the TNF-α/IL10 index caused by LPS.

It can be inferred that the neuroprotective activities described for SC and HR contribute to the therapeutic effect among the different doses of treatment with the active fraction of standardized in coumarins of *T. lucida*. However, the expectation arises that DF also contributes to this effect due to two considerations: the first, due to the structural similarity of SC, HR, and DF (pharmacodynamic approach) and, on the other hand, that DF has the highest bioavailability index among the coumarins present in the bioactive fraction. For this reason, this study is part of the technological development of a neuroprotective medication based on the standardized extract of *T. lucida*, where it is defined that both the 10 and 20 mg/kg doses are effective in counteracting the damage caused by neuroinflammation underlying the administration of LPS, protecting the BBB, and generating an immunomodulatory environment.

## Figures and Tables

**Table 1:** Identification parameters of coumarins by HPLC-UV.

Analyte	Concentration (µg/mL)	TR (min)	λ_max1_ (nm)	λ_max2_ (nm)	λ_max3_ (nm)
PE	165.9 ± 1.49	9.826	219.2	324.4	---
SC	225.9 ± 3.21	11.351	229.8	294.7	343.4
DF	234.6 ± 8.60	12.844	215.7	295.9	338.6
HR	203.0 ± 3.57	13.689	219.2	323.2	---
PU	103.8 ± 0.41	28.051	204.0	323.2	---

PE = 7-*O*-prenylscopoletin, SC = scoparone, DF = dimethylfraxetin, HR = herniarin, and PU = 7-*O*-prenylumbelliferone. Results are presented as mean ± S.D. (*n* = 3).

**Table 2:** Pharmacokinetic parameters of coumarins estimated by non-compartmental analysis in ICR mice plasma after different doses of oral administration of bioactive fraction.

**Dose = 5 mg/kg**						
**Parameter**	**Unit**	**PE**	**SC**	**DF**	**HR**	**PU**
C_max_	µg/mL	0.35 ± 0.05	0.65 ± 0.02	0.58 ± 0.11	0.71 ± 0.09	0.38 ± 0.07
T_max_	h	1.05 ± 0.40	1.55 ± 0.34	0.50 ± 0.25	0.45 ± 0.12	0.80 ± 0.20
t_1/2_	h	2.76 ± 0.49	4.62 ± 2.47	3.10 ± 0.60	4.03 ± 1.94	3.76 ± 1.12
AUC_0–t_	µg·h/mL	0.77 ± 0.08	2.38 ± 0.13	1.28 ± 0.17	1.55 ± 0.21	1.14 ± 0.09
AUC_0–∞_	µg·h/mL	1.10 ± 0.22	4.45 ± 1.30	1.92 ± 0.50	2.25 ± 0.26	1.71 ± 0.32
MRT	h	4.29 ± 0.98	7.62 ± 3.51	4.66 ± 0.88	6.04 ± 2.42	5.53 ± 1.69
CL/F	*	53.20 ± 9.97	13.99 ± 2.51	30.94 ± 4.76	23.71 ± 3.26	32.81 ± 5.01
Vd/F	&	184.67 ± 9.15	59.25 ± 15.66	130.15 ± 30.65	122.40 ± 46.54	149.73 ± 14.28
Dose = 10 mg/kg [21]						
Parameter	Unit	PE	SC	DF	HR	PU
C_max_	µg/mL	1.77 ± 0.31	1.28 ± 0.11	1.82 ± 0.27	1.48 ± 0.19	0.69 ± 0.07
T_max_	h	0.85 ± 0.28	0.70 ± 0.34	0.60 ± 0.24	0.25 ± 0.00	1.05 ± 0.31
t_1/2_	h	4.48 ± 1.31	0.65 ± 0.06	1.50 ± 0.37	1.49 ± 0.35	0.95 ± 0.10
AUC_0–t_	µg·h/mL	3.52 ± 0.10	2.77 ± 0.21	3.25 ± 0.31	2.95 ± 0.28	2.22 ± 0.26
AUC_0–∞_	µg·h/mL	6.37 ± 1.20	2.78 ± 0.21	3.61 ± 0.40	3.21 ± 0.38	2.24 ± 0.26
MRT	h	6.96 ± 1.92	2.26 ± 0.17	2.72 ± 0.30	2.77 ± 0.25	2.68 ± 0.14
CL/F	*	17.38 ± 2.25	36.52 ± 2.68	28.81 ± 2.51	32.50 ± 2.91	46.95 ± 5.03
Vd/F	&	95.74 ± 8.76	35.34 ± 5.14	58.00 ± 8.77	66.54 ± 14.92	66.62 ± 12.38
Dose = 20 mg/kg						
Parameter	Unit	PE	SC	DF	HR	PU
C_max_	µg/mL	1.96 ± 0.10	2.02 ± 0.14	3.13 ± 0.20	1.80 ± 0.20	0.88 ± 0.06
T_max_	h	0.25 ± 0.00	1.35 ± 0.15	0.75 ± 0.00	0.45 ± 0.12	1.50 ± 0.31
t_1/2_	h	4.30 ± 0.56	2.57 ± 0.80	1.63 ± 0.10	2.69 ± 0.59	1.70 ± 0.18
AUC_0–t_	µg·h/mL	6.76 ± 0.48	6.22 ± 0.22	8.33 ± 0.52	3.71 ± 0.11	3.21 ± 0.30
AUC_0–∞_	µg·h/mL	10.84 ± 1.08	7.91 ± 0.79	8.87 ± 0.54	5.07 ± 0.56	3.55 ± 0.25
MRT	h	6.27 ± 0.84	4.07 ± 0.90	2.63 ± 0.12	4.21 ± 0.85	3.15 ± 0.15
CL/F	*	19.20 ± 1.88	26.17 ± 2.25	22.90 ± 1.44	41.34 ± 4.26	57.62 ± 4.71
Vd/F	&	115.48 ± 10.99	87.06 ± 20.06	53.49 ± 3.08	146.80 ± 20.34	143.61 ± 21.95

Concentration quantification after oral dose administration of 5, 10 [21], or 20 mg/kg. Values represent mean ± SEM (*n* = 5). PE = 7-*O*-prenylscopoletin, SC = scoparone, DF = dimethylfraxetin, HR = herniarin, and PU = 7-*O*-prenylumbelliferone. * (mg/kg)/(μg/mL)/h. & (mg/kg)/(μg/mL).

## Data Availability

The data presented in the study is available in the article.

## Supplementary Materials

The following supporting information can be downloaded at: https://www.mdpi.com/article/10.3390/pharmaceutics15030967/s1, Table S1: Elution gradient of the analytical method by HPLC-UV for coumarin determination in a bioactive fraction of *T. lucida*.
