# Supplementary material for: Dose-Effect Determination of a Neuroprotector Fraction Standardized in Coumarins of Tagetes lucida and Bioavailability
Source: Pharmaceutics. 2023 Mar 17;15(3):967. doi: 10.3390/pharmaceutics15030967 (PMC10051064; doi:10.3390/pharmaceutics15030967)
Supplement: Supplementary file 1 [file pharmaceutics-15-00967-s001.zip › pharmaceutics-2242920-supplementary.pdf]

pharmaceutics

MDPI

Supplementary Materials

Dose-effect determination of a neuroprotector fraction standardized in coumarins of *Tagetes lucida* and bioavailability.

Anislada Santibáñez [1, 2], Maribel Herrera-Ruiz [1], Manasés González-Cortazar [1], Pilar Nicasio-Torres [1], Ashutosh Sharma [2, *], and Enrique Jiménez-Ferrer [1, *]

1 Centro de Investigación Biomédica del Sur, Instituto Mexicano del Seguro Social, Argentina No. 1 Col Centro, Xochitepec Morelos 62790, Mexico
2 School of Engineering and Sciences, Tecnologico de Monterrey, Av. Epigmenio González No. 500, San Pablo, Queretaro 76130, Mexico
* Correspondence: asharma@tec.mx (A.S.); enriqueferrer_mx @yahoo.com (E.J.-F.)

Table S1. Elution gradient of the analytical method by HPLC-UV for coumarin determination in a bioactive fraction of *T. lucida*

Time (min)	% A	% B
0.00	100.00	0.00
1.00	100.00	0.00
2.00	95.00	5.00
3.00	95.00	5.00
4.00	70.00	30.00
20.00	70.00	30.00
21.00	50.00	50.00
23.00	50.00	50.00
24.00	20.00	80.00
25.00	20.00	80.00
26.00	0.00	100.00
27.00	0.00	100.00
28.00	100.00	0.00
30.00	100.00	0.00

% A: Aqueous solution of trifluoroacetic acid at 0.5%; %B: acetonitrile.
